# Discovery of Anti-Hypertensive Oligopeptides from Adlay Based on In Silico Proteolysis and Virtual Screening

**DOI:** 10.3390/ijms17122099

**Published:** 2016-12-14

**Authors:** Liansheng Qiao, Bin Li, Yankun Chen, Lingling Li, Xi Chen, Lingzhi Wang, Fang Lu, Ganggang Luo, Gongyu Li, Yanling Zhang

**Affiliations:** Key Laboratory of TCM Foundation and New Drug Research, School of Chinese Material Medica, University of Chinese Medicine, Beijing 100102, China; b20100222012@163.com (L.Q.); libinzyy@163.com (B.L.); 18811791975@163.com (Y.C.); lilingling94@126.com (L.L.); chenxi_cx95@163.com (X.C.); wanglz@bucm.edu.cn (L.W.); lufang1017@163.com (F.L.); 17801080765@163.com (G.Lu.); lidoc2727@163.com (G.Li)

**Keywords:** adlay, hypertension, oligopeptides, angiotensin-I converting enzyme (ACE), in silico proteolysis, molecular dynamics (MD)

## Abstract

Adlay (*Coix larchryma-jobi* L.) was the commonly used Traditional Chinese Medicine (TCM) with high content of seed storage protein. The hydrolyzed bioactive oligopeptides of adlay have been proven to be anti-hypertensive effective components. However, the structures and anti-hypertensive mechanism of bioactive oligopeptides from adlay were not clear. To discover the definite anti-hypertensive oligopeptides from adlay, in silico proteolysis and virtual screening were implemented to obtain potential oligopeptides, which were further identified by biochemistry assay and molecular dynamics simulation. In this paper, ten sequences of adlay prolamins were collected and in silico hydrolyzed to construct the oligopeptide library with 134 oligopeptides. This library was reverse screened by anti-hypertensive pharmacophore database, which was constructed by our research team and contained ten anti-hypertensive targets. Angiotensin-I converting enzyme (ACE) was identified as the main potential target for the anti-hypertensive activity of adlay oligopeptides. Three crystal structures of ACE were utilized for docking studies and 19 oligopeptides were finally identified with potential ACE inhibitory activity. According to mapping features and evaluation indexes of pharmacophore and docking, three oligopeptides were selected for biochemistry assay. An oligopeptide sequence, NPATY (IC_50_ = 61.88 ± 2.77 µM), was identified as the ACE inhibitor by reverse-phase high performance liquid chromatography (RP-HPLC) assay. Molecular dynamics simulation of NPATY was further utilized to analyze interactive bonds and key residues. ALA354 was identified as a key residue of ACE inhibitors. Hydrophobic effect of VAL518 and electrostatic effects of HIS383, HIS387, HIS513 and Zn^2+^ were also regarded as playing a key role in inhibiting ACE activities. This study provides a research strategy to explore the pharmacological mechanism of Traditional Chinese Medicine (TCM) proteins based on in silico proteolysis and virtual screening, which could be beneficial to reveal the pharmacological action of TCM proteins and provide new lead compounds for peptides-based drug design.

## 1. Introduction

Traditional Chinese Medicines (TCMs) are commonly used natural products, and some of them have high protein content. However, pharmacological mechanism exploration of these TCMs is always difficult because of the isolation difficulty of proteins. Actually, the prejudice that primary metabolites, including proteins and nucleic acids, have the low bioactivities is also not conducive to the study on pharmacological mechanism of TCM proteins. With in-depth research of protein and peptide drugs, the pharmacological activity of TCM-derived proteins is also gradually addressed by researchers. Especially, many oligopeptides or peptide fractions are discovered from animal drugs of TCM, and the studies on their biological activities are usually guided based on the traditional pharmacological effect. For instance, two hematopoietic peptides were identified from *Colla corii asini* by Wu [1] and three antioxidant peptides were obtained from *Cornu bubali* by Liu [2]. Compared with the animal drugs from TCM, the research of TCM proteins from herbal medicine seems to be ignored. Instead, plenty of oligopeptides and peptide fractions have been identified from non-medicinal plants, such as *Sorghum bicolor* [3], *Pennisetum glaucum* [4], and so on. However, the studies on the bioactivities of these plant peptides are often random, due to the lack of traditional pharmacological effect like TCM.

Adlay (*Coix larchryma-jobi* L.) is a commonly used TCM for the clinical treatment of cardiovascular diseases (CVD) and hypertension [5]. Adlay has high protein content, including albumins, globulins, prolamins and glutelins. Prolamins accounted for nearly 60% of adlay seed storage proteins [6]. Adlay protein and peptides have various pharmacological actions, including CVD and hypertension [7,8]. Previous research has proven that bioactive peptides released by enzymatic digestion were effective components of adlay for hypertension and ACE inhibiting [9]. However, the structures and anti-hypertensive mechanism of adlay bioactive oligopeptides are not clear. It is necessary to discover novel oligopeptides from adlay and illustrate the anti-hypertensive mechanism.

Natural proteins could be digested and divided into peptides by digestive tract enzymes and intestinal flora. It is an essential source of oligopeptides from TCM and foods, which were further absorbed into blood circulation and contributed to treating diseases. With the development of sequencing technology and proteomics, increasing protein sequences of TCM and foods were revealed. Meanwhile, various methods were also developed for in silico proteolysis to analyze the potential cleavage sites of protein sequences, such as BIOPEP [10] and PeptideCutter [11], which can be utilized to in silico hydrolyze known protein sequences, discover novel oligopeptides, and illuminate the action mechanism of natural proteins. Compared to in vivo proteolysis, the accuracy of in silico proteolysis has been validated by multiple approaches, including liquid chromatograph-mass spectrometer (LC-MS) [10,12,13,14]. In silico proteolysis is widely used to analyze natural proteins and generate bioactive peptides from food and halobios, such as milk, meat, tilapia, sea cucumber and so on [15,16,17,18]. A biological assay indicated the reliability of in silico proteolysis, which could be further used for other types of natural products or TCM.

In this paper, systematic identification of adlay bioactive peptides was implemented by in silico proteolysis and virtual screening. According to the sequences of adlay prolamins, in silico proteolysis was carried out based on the simulated gastrointestinal environments with three typical digestive enzymes. Adlay oligopeptides obtained by in silico proteolysis were used to construct the oligopeptide library. Then, anti-hypertensive pharmacophore profiling was used to discover the targets of oligopeptides by anti-hypertensive pharmacophore database, and ACE was identified as the potential target for anti-hypertensive activity. The pharmacophore screening results were further refined by molecular docking. The docking scores and high frequency residues were regarded as the assessment criteria to identify the potential ACE inhibitors (ACEIs). Biochemistry assay and molecular dynamics (MD) simulation of potential ACEIs were carried out to validate predicted bioactivities and binding stability between oligopeptides and ACE. This study provides a new research mode for revealing the action mechanism of TCM proteins. Combining in silico proteolysis, molecular simulation and biochemistry assay, TCM oligopeptides could be obtained and used to discover their bioactivities rapidly.

## 2. Results

### 2.1. Adlay Oligopeptides Database

**Figure 1:**
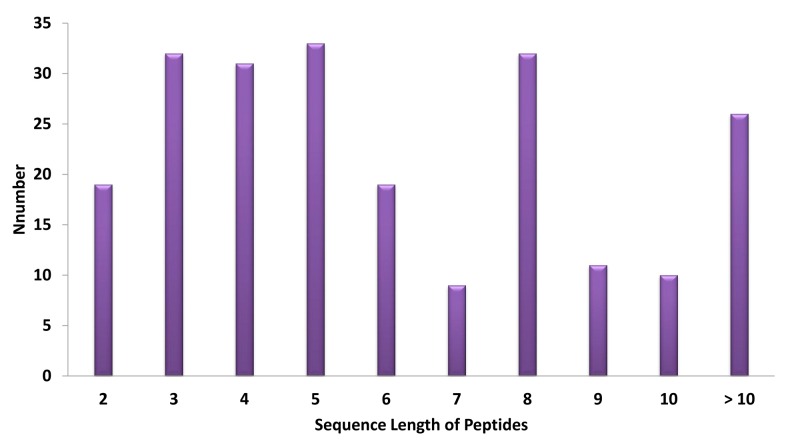
Distribution of the peptide library of adlay.

According to in silico gastrointestinal digestion proteolysis, 222 peptides were obtained and their sequence distribution is shown in Figure 1. Wherein, 134 oligopeptides (60%) were distributed from dipeptide to hexapeptide and utilized to construct the oligopeptides library, because these oligopeptides have reasonable molecular weight, absorption properties and druggability. Compared to the sequences between simulated proteolysis results of adlay and the BIOPEP active oligopeptides database, 11 known active oligopeptides were consistent and are shown in Table 1. Their main bioactivities were ACE inhibition, antioxidation and glucose uptake stimulation. Especially for the ACE inhibitory peptides, hundreds of ACE inhibitory peptides were reported and recorded from multiple species in the BIOPEP, but none came from adlay. According to the research of in silico proteolysis of adlay, ten known ACEIs from oligopeptides library were obtained from Reference [19,20]. However, Bioactivity of more than 90% of oligopeptides from adlay prolamin was not clear and need further study.

### 2.2. Reverse Target Identification by Pharmacophore

Pharmacophore is an important method to reverse identify action targets for candidate compounds [23], and is widely used for drug repositioning [24] and polypharmacology prediction of TCM [25]. In this study, the library of 134 oligopeptides was used to reverse search for action targets based on pharmacophore. The screening results are shown in Table 2. According to the analysis of hit number of adlay oligopeptides for every hit target, ACE, ATP-sensitive potassium channel and β-adrenoceptor might be the most important targets for adlay oligopeptides. At the same time, fit value was also an important judgment index and needs to be further considered for pharmacophore screening, representing the overlap degree between oligopeptides and model [26]. ACE had obvious advantages compared to other targets, as ACE not only had the largest hit number of adlay oligopeptides, but also had the largest hit number of adlay oligopeptides with their fit values more than 0.7, which indicated that oligopeptides had the superior predicted activity of ACE. Therefore, ACE was regarded as the main potential target for the anti-hypertensive activity of adlay oligopeptides, which was further used to discover the anti-hypertensive oligopeptides from adlay in this paper. Actually, the biological activity of the oligopeptides against other target should also be studied in the future research.

As for the ten known ACEIs from oligopeptides library in Table 1, eight oligopeptides were hit by ACE pharmacophore model, which indicated the reliability of ACE pharmacophore and screening technology. ACE pharmacophore model was constructed by training set of seven ACEIs (Figure S1), as shown in Figure S2A. This 3D quantitative pharmacophore hypothesis contained four features: one hydrogen bond acceptor (A), one hydrogen bond donor (D), one negative ionizable group (N) and one hydrophobic group (H). A set of 41 oligopeptides with fit values more than 0.7 were obtained based on virtual screening of ACE pharmacophore model. However, ACE pharmacophore in this paper was constructed by ligand-based method and did not consider the structure of protein, which could result in high false positive rate during screening. In order to further refine the screening results of adlay oligopeptides for ACE inhibitory activity, 41 hit oligopeptides were further docked into ACE crystal structures.

### 2.3. Docking Analysis of ACE Inhibitors 

**Figure 2:**
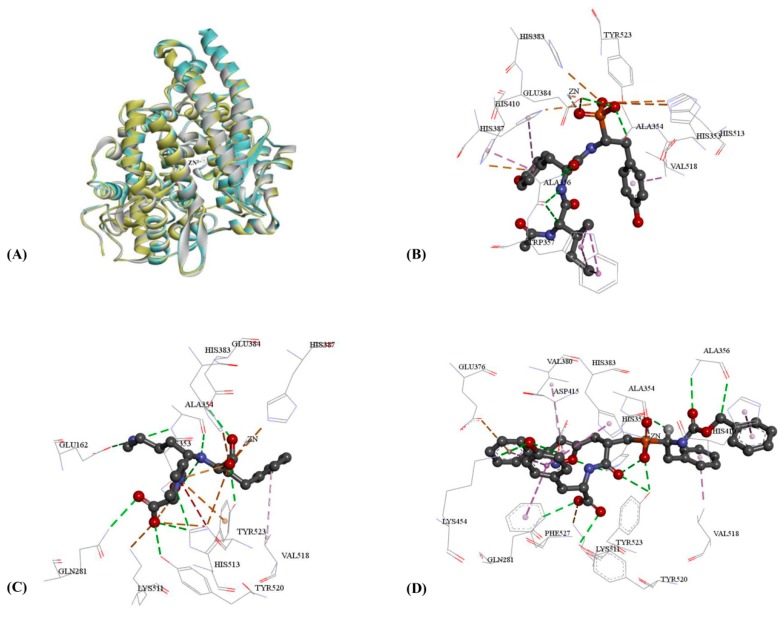
The 3D docking results of initial ligands and three crystal structures: (**A**) the alignment of three crystal structures with 1O86 in blue, 4BZR in grey, and 4CA in yellow; (**B**) docking results of 4BZR and K-26; (**C**) docking results of 1O86 and lisinopril; and (**D**) docking results of 4CA5 and FI. The pink dash line represented the hydrophobic effect. The green dash line represented the hydrogen bond donor. The orange dash line represented the electrostatic network.

According to the reverse target identification, ACE was found to be the potential target for the anti-hypertensive activities of adlay. The molecular docking algorithm of CDOCKER was used to further refine the pharmacophore screening results. CDOCKER is a semi-flexible docking algorithm, and the protein is held rigid [27,28]. In the process of semi-flexible docking, the active site of protein kept the conformation which was induced by ligand. Therefore, CDOCKER might cause high false positive rate based on one crystal structure induced by ligand. In order to improve the accuracy of docking and reduce the false positive rate, three crystal structures with different initial ligands and reasonable resolutions from RCSB Protein Data Bank (PDB) were used for docking study. 1O86 was the first revealed C domain structure of ACE, and the initial ligand was the approved drug of lisinopril [29]. This crystal structure could better predict the ACE bioactivities and was widely used in various docking studies of ACE [30,31]. The initial ligand of 4CA5 was specific phosphinic tripeptide named FI [32]. Using 4CA5 might be beneficial to screen the oligopeptides because the similar chemical structure might perform the similar binding mode in protein. Meanwhile, 4BZR combined with N-modified tripeptide named K-26 was the latest revealed crystal structure and the highest resolution, which might provide more precise details of ACE and oligopeptides [33]. The proteins of three crystal structures of 1O86, 4CA5 and 4BZR were aligned to analyze the difference of protein conformations, and Zn^2+^ was also overlapped well in active sites (shown in Figure 2A). According to TM-align analysis [34], the pairwise root mean-square deviation (RMSD) between three protein conformations were all less than 0.35 Å, and TM-scores were all higher than 97%, which displayed slight difference between three protein conformations.

Three crystal structures were further used for docking study, and the details of the docking models are shown in Table 3. RMSD values among three re-docked ligands and crystal structures were all less than 2.00 Å, which indicated that the docking algorithm CDOCKER and docking parameters were applicable to these crystal structures. The radiuses of active sites in three crystal structures were different because of the difference of molecular weight for initial ligands. 4BZR had the smallest radius, while 4CA5 had the largest radius. The docking models with different sphere radius might be beneficial for the distinguishing potential active oligopeptides. –CDOCKER ENERGY (kcal/mol) was the scoring function of CDOCKER algorithm, which represented the energy of the ligand–receptor complexes [35]. Fifty percent of –CDOCKER ENERGY for three crystal structures were regarded as the threshold for screening the potential ACE inhibitory oligopeptides [36,37].

The interactions of initial ligands with 4BZR (Figure 2B), 1O86 (Figure 2C), and 4CA5 (Figure 2D) were analyzed for identifying binding residues. Their interactive residues and structural features were obtained based on initial ligands (Table S1). In the 3D docking results of three crystal structures, all initial ligands could perform hydrogen bond interactions with HIS353, TYR523, and ALA354. Electrostatic effect with Zn^2+^ and hydrophobic effect with VAL518 were also common binding interactions with ACE for three crystal structures, which indicated these residues and Zn^2+^ might be the key binding interaction for ACE inhibitory activity. 1O86, HIS383 and LYS511 could form salt bridges with two carboxyl groups, and HIS383 also formed the salt bridge for 4BZR. In order to analyze the difference of docking models and identify a more representative model, docking results of 1O86, 4CA5 and 4BZR were compared in pairs (Table 4). Similar interactions of binding residues were displayed between three crystal structures and their initial ligands.

According to the docking results of 13 positive ACEIs, they were all successfully docked into three active pockets, which was regarded as the first principle for screening the competitive ACE inhibitory oligopeptides [38]. Every positive ACEI could form 10 poses in one crystal structure. Therefore, 13 positive ACEIs for every crystal structure could form 130 poses (Table S1). The interactive residues were extracted from every pose, and then the residue results of 130 poses were counted and ranked for identifying high frequency residues. The top four residues for every crystal structure were regarded as the high frequency residues. According to analysis of high frequency residues from three crystal structures, the hydrogen bond interaction with ALA354 and hydrophobic interaction with VAL518 were identified as the consistent interaction for positive ACEIs. It also indicated that docking poses of three crystal structures had some structural differences. Actually, Zn^2+^ was always the key role forming the metal-acceptor interaction. However, electrostatic interaction seemed not to be quite important for the interaction of positive ACEIs and ACE, which appeared low frequency in docking process.

According to docking models from three crystal structures, 41 hits oligopeptides were further refined to identify the potential ACEIs. A set of 24 oligopeptides was obtained in docking process by 4BZR. Two sets of 34 and 38 oligopeptides were also obtained by 1O86 and 4CA5, respectively. Although all 13 positive ACEIs have been docked into three active pockets successfully, a total of 19 oligopeptides from adlay docked into three active pockets successfully, which indicated that combination screening was essential based on multiple crystal structures. As the potential ACEIs of 19 oligopeptides, their interactive residues were also analyzed for selecting the reasonable oligopeptides for further biochemistry assay. The analysis of high frequency residues between 19 oligopeptides and three crystal structures is shown in Table S1 and the pairwise comparison of high frequency residues is shown in Table 4. According to comparison of three lists of high frequency residues from three crystal structures, no same high frequency residue was discovered in all three crystal structures with 19 oligopeptides, which was related to false positives of screening and the difference of radius of active site. Although some differences of the interaction appeared in three crystal structures, 1O86 could also indicate the partial consistency with 4BZR or 4CA5, respectively, as shown in Table 4. 1O86 had more same high frequency residues with 4BZR or 4CA5, but 4BZR and 4CA5 showed less similarity with each other. Therefore, 1O86 with the intermediate radius could be a better representation of ACE crystal structure, which was further utilized in MD studies. However, scoring functions, which lacked the information of key residues, were one-sided evaluation for screening results. In this paper, high frequency residues between 1O86 and ACEIs were also considered to select potential oligopeptides for biological assay. Three oligopeptides were regarded as possible lead candidates of ACEIs with different interaction of ALA354 and VAL518, including NPATY, NCHEF, and VSAIGF (Table 5). NPATY could interact with both two high frequency residues. VSAIGF could only interact with VAL518 and NCHEF could only produce hydrogen bond interaction with ALA354. Actually, this selection method might exist the contingency based on high frequency residues, since docking result was only the time point of the interactive residues between oligopeptides and ACE. Therefore, screening results of all 19 oligopeptides are displayed in Table S2 for future research, and biological assay and MD study would be further implemented for three oligopeptides.

### 2.4. Biological Evaluation of ACEIs

**Figure 3:**
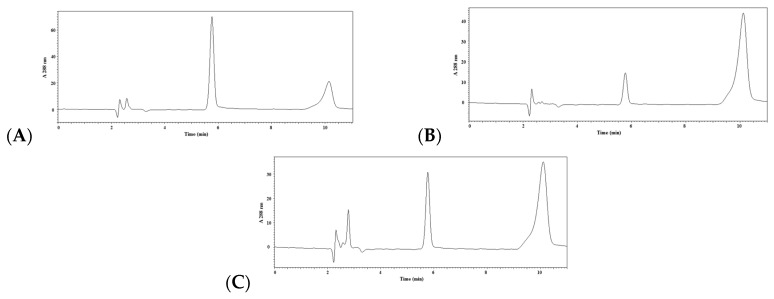
RP-HPLC chromatograms of: blank control (**A**); lisinopril at 2 × 10^−9^ mol/L (**B**); and NPATY at 0.048 mg/mL (**C**) detected at 228 nm. Hippuric acid (HA) and hippuryl-l-histidyl-l-leucine (HHL) standard eluted out at 5.5 and 9.2 min.

The reverse-phase HPLC (RP-HPLC) method was utilized to estimate ACE inhibitory activity. Hippuric acid (HA) and hippuryl-l-histidyl-l-leucine (HHL) standard eluted out of the C_18_ column at 5.5 and 9.2 min, respectively (Figure S3), and were well separated in assay mixture (Figure 3A). The control had no inhibitory activity and thus displayed a strong peak area of HA, while lisinopril (2 × 10^−9^ mol/L) manifested a strong ACE inhibition ratio of 77.86% ± 0.15% (Figure 3B). Then, the ACE inhibitory activities of three oligopeptides were evaluated by RP-HPLC, including NPATY, NCHEF, and VSAIGF. The HPLC chromatograms of NPATY, NCHEF, and VSAIGF with 0.048 mg/mL are shown in Figure 3C and Figure S3. Only NPATY acted as an ACE inhibitor with an IC_50_ of 61.88 ± 2.77 μM. The results implied that the observed inhibitory effects of NPATY, although not as powerful as lisinopril.

**Figure 4:**
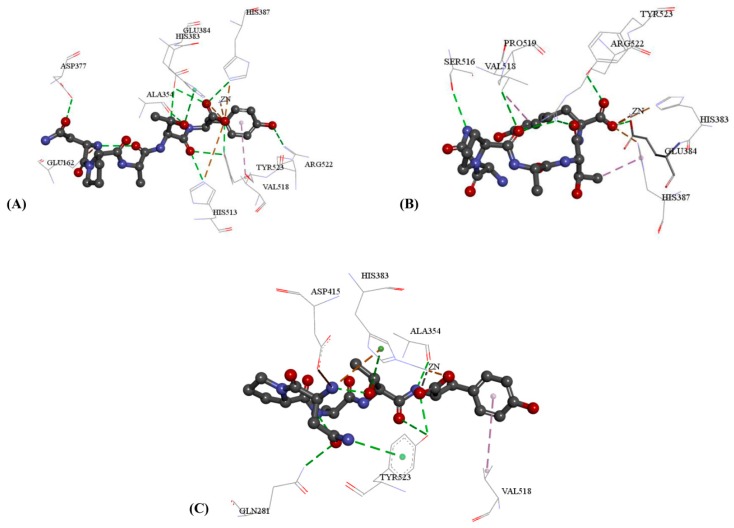
The 3D key interaction between NPATY and three crystal structures: (**A**) 1O86 and NPATY; (**B**) 4BZR and NPATY; and (**C**) 4CA5 and NPATY. Wherein, green dash lines represent hydrogen bond; orange dash lines represent electrostatic interaction; and pink dash lines represent hydrophobic effect.

**Figure 5:**
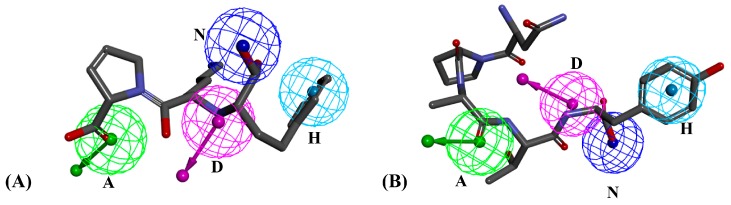
Matching graphs of ACE pharmacophore model and ligands: Lisinopril matched pharmacophore with fit value of 0.947 (**A**); and NPATY matched pharmacophore with fit value of 0.969 (**B**). Wherein, green features represent hydrogen bond acceptor; pink features represent hydrogen bond donor; dark blue features represent negative ionizable group; and light blue features represent hydrophobic group.

**Figure 6:**
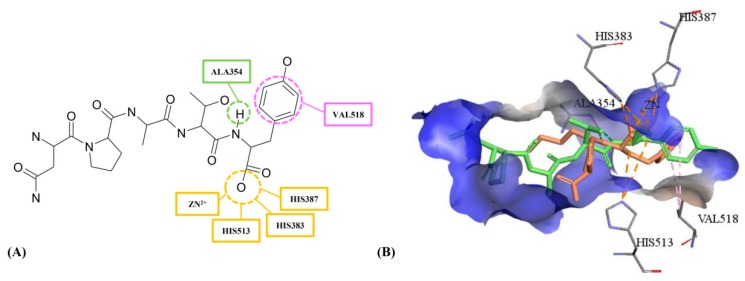
The 2D and 3D interaction analysis of NPATY based on the common features between pharmacophore and docking models: (**A**) 2D common interaction of NPATY included hydrophobic bonds with VAL518, hydrogen bond with ALA354, and electrostatic effects with HIS383, HIS387, HIS513 and Zn^2+^; and (**B**) 3D common interaction of lisinopril (orange) and NPATY (green). The pink dash line represent the hydrophobic effect; the green dash line represent the hydrogen bond donor; and the orange dash line represent the electrostatic network.

As the proven ACE inhibitory activity, NPATY was the active inhibitor and its interaction with three crystal structures are displayed in Figure 4. NPATY mapped all four features with ACEI pharmacophore with fit value of 0.969 (Figure 5B). NPATY obtained a higher docking score in three crystal structures and could form three types key interaction at the 1O86 active site: (1) a hydrogen bond network with the residues HIS383, GLU384, HIS387, HIS513, ARG522, TYR523, ALA354, ASP377, and GLU376; (2) electrostatic interaction with GLU162, HIS383, HIS387, HIS513, and Zn^2+^; and (3) hydrophobic interactions with VAL518. Hydrogen bond, electrostatic and hydrophobic effects were the main ligand–enzyme interactions, which were consistent with the pharmacophore results of present study, and provided more structural information of ACE peptide inhibitors (Figure 6). C-terminal of ACE inhibitory oligopeptides might be more important to ACE inhibitory oligopeptides because the pharmacophore results indicated that four features were all matched with C-terminal of oligopeptides. Benzene ring of tyrosine in C-terminal of NPATY could form hydrophobic effect with VAL518 and also map with hydrophobic feature in pharmacophore. It indicated VAL518 might be the key residue for ACEIs, which has also been proven by mutation analysis [39]. Meanwhile, hydroxyl oxygen on carboxyl group in C-terminal of NPATY could also preform the electrostatic network with HIS383, HIS387, HIS513 and Zn^2+^ and map with negative ionizable group in pharmacophore. The hydrogen atom on the first acyl-nitrogen from the C-terminal of NPATY could produce hydrogen bond effect with ALA354 and map with hydrogen bond donor in pharmacophore. ALA354 has also been identified as the key residue for oligopeptides by Wu [40] and Jimsheena [41]. As shown in Figure 6, NPATY showed consistency with docking and pharmacophore model and shared a similar binding mode with lisinopril.

However, NCHEF and VSAIGF could not obtain the desirable activities of ACE. The pharmacophore mapping graphs of NCHEF and VSAIGF are shown in Figure S2 and their docking results are displayed in Table S1. The conformation of VSAIGF produced the fold when it mapped with the pharmacophore model, so the pharmacophore features matched with both C-terminal and N-terminal. Meanwhile, the pharmacophore mapping of NCHEF produced the deviation that hydrogen donor and negative ionizable features matched with the second amino acids rather than the first amino acids from C-terminal of NCHEF. As for the docking analysis, VSAIGF could not form the hydrogen bond interaction with ALA354, and NCHEF could not produce the hydrophobic bonds with VAL518, which might be the reason of the low ACE activity.

### 2.5. Molecular Dynamics Simulation

**Figure 7:**
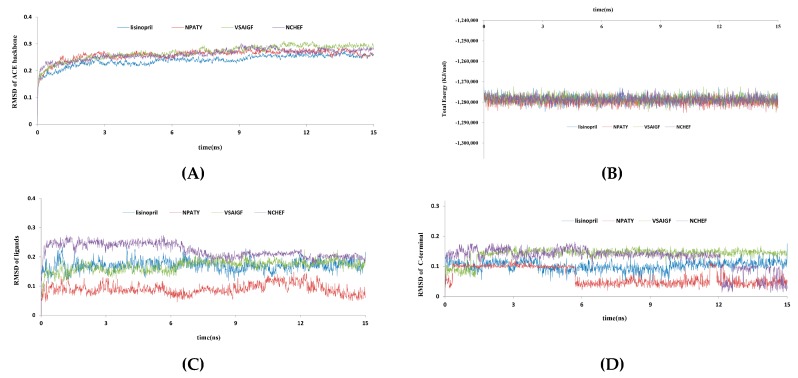
Trajectory of the MD simulation for four complexes: (**A**) average protein backbone RMSD; (**B**) total Energy; (**C**) RMSD of ligands; and (**D**) RMSD of first active amino acids in C-terminal of ligands. Blue indicates lisinopril–1O86; red indicates NPATY–1O86; green indicates VSAIGF–1O86; and purple indicates NCHEF–1O86.

Molecular docking studies are always limited by vacuum environment and semi-flexible methods, which could result in false positive and deviation of key residues. Therefore, molecular dynamics (MD) simulation was implemented to validate the docking results and evaluate the stability of ACE-inhibitor complexes under dynamic conditions in this paper. Four initial complexes conformations of 1O86–lisinopril, 1O86–NPATY, 1O86–NCHEF and 1O86–VSAIGF were acquired from the molecular docking of CDOCKER. The RMSD trajectories of ACE protein backbone and total energy profiles of each complex are shown in Figure 7A,B. The RMSD and total energy of four systems were stabilized within 15 ns, and the trajectories of complexes fluctuated slightly after 3 ns and reached equilibrium after 6 ns. The NPATY had similar total energy property to lisinopril.

**Figure 8:**
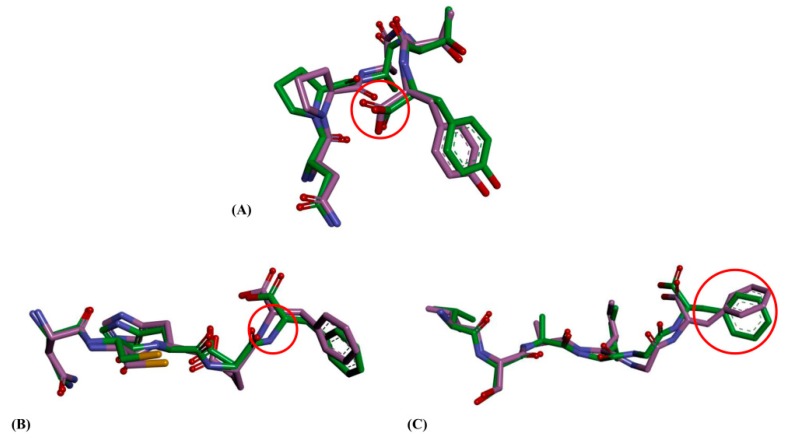
Conformational changes of first active amino acids in C-terminal of ligands: (**A**) conformational changes of NPATY from 5.70 to 5.71 ns; (**B**) conformational changes of VSAIGF from 1.39 to 1.40 ns; and (**C**) conformational changes of NCHEF from 12.05 to 12.06 ns. The conformational changes were from green conformation to pink conformation. The red circles represent the important differences in active amino acids.

The RMSD of four ligands was also analyzed and are displayed in Figure 7C. The four ligands could reach to relative equilibrium state after 8 ns. However, the RMSD of first amino acids in C-terminal of ligands could not keep equilibrium during 15 ns, except lisinopril. According to the results of pharmacophore and docking, the first active amino acids in C-terminal of ligands were discovered to play an important role to ACEIs. Therefore, the conformational changes of the first active amino acids in C-terminal of three oligopeptides were further analyzed from MD process (Figure 8) [42,43]. The conformational change of NPATY was extracted from 5.70 to 5.71 ns. The carboxyl group in C-terminal of NPATY rotated nearly 180°, but it might produce a slight influence to the electrostatic effect based on the property of carboxyl group. The conformational change of VSAIGF was extracted from 1.39 to 1.40 ns, which showed the large torsion of the N atom in phenylalanine. It might affect the interaction of ALA354. The conformational change of NCHEF was also extracted from 12.05 to 12.06 ns. Large torsion fluctuations observed for NCHEF were the benzene ring of phenylalanine in C-terminal, which might affect the hydrophobic effect with VAL518.

**Figure 9:**
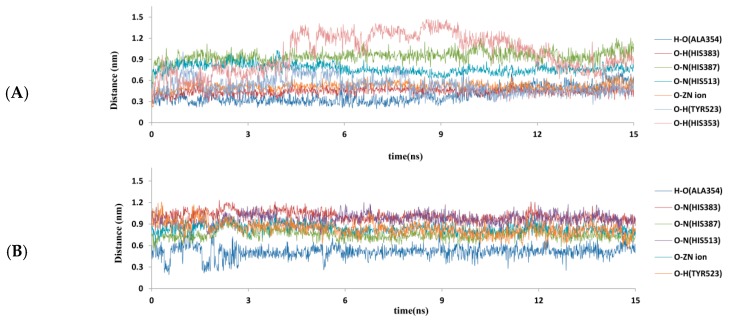
Time dependent atom distances (nm) for: lisinopril (**A**); and NPATY (**B**) in the 1O86 binding site during 15 ns MD simulation.

According to the results of pharmacophore and docking, ALA354, HIS383, HIS387, HIS513 and Zn^2+^ were crucial to the ACE inhibitory activity and beneficial to the stability of complexes. Therefore, the atom distances of 1O86–lisinopril and 1O86–NPATY were further analyzed during the simulation time (Figure 9A,B). The atom distances fluctuated slightly after 3 ns and reached equilibrium after 6 ns. As for the electrostatic network of HIS383, HIS387, HIS513 and Zn^2+^ with ligands, lisinopril and NPATY could reach equilibrium after 6 ns. It indicated electrostatic interaction might be important to ACE inhibition. Compared with TYR523 and HIS353, ALA354 could keep equilibrium for lisinopril and NPATY after 6 ns, which was consistent with results of pharmacophore and docking. ALA354 might be the key residues for ACEI. MD provided water environment and flexible dynamic conditions, which was beneficial to analyze the stability of key residues. Actually, MD simulation ignored the roles of the electron, so the confirmation of key residues also needed to rely on further mutation studies and quantum mechanical analysis.

## 3. Materials and Methods

### 3.1. In Silico Proteolysis of Adlay Prolamin

The protein sequences of 10 adlay prolamin were identified by LIN, including α-coixin 1-8, γ-coixin, and Δ-coixin [6]. In order to analyze the activity of adlay oligopeptides, in silico proteolysis was implemented to predict the hydrolysis sites of coixins by a dedicated tool in the BIOPEP database [10]. Wherein, three typical enzymes of pepsin (pH > 1.3), trypsin and chymotrypsin in the gastrointestinal digestion tract were utilized for in silico analysis [16,17]. Following in silico digestion, the generated peptides were also compared with previous bioactive peptides in BIOPEP. Released oligopeptides composed of 2 to 6 amino acids with reasonable molecular weight were selected and utilized to construct the virtual peptide library by Discovery Studio 4.0 (DS). Because of the study on natural oligopeptides, L-amino acids were chosen as the assembled unit of oligopeptides. All oligopeptides were generated 3D structures and full minimized in CHARMm force field with MMFF94 partial charge.

### 3.2. Anti-Hypertensive Pharmacophore Profiling

Ligand profiler module was utilized to reversely identify targets of oligopeptides from adlay [25]. Initially, diverse conformations of oligopeptides were generated by BEST mode with 255 conformations, and the relative energy threshold was less than 20.0 kcal/mol. The generated results were regarded as query to screen the anti-hypertensive pharmacophore database by the flexible searching method. This anti-hypertensive database was built by long-term studies of our group and contained 10 commonly used anti-hypertensive targets, including ACE, angiotensin II type 1 receptor (AT1), endothelin A receptor (ETA), endothelin B receptor (ETB), neutral endopeptidase (NEP), mineralocorticoid receptor (MR), renin (REN), β-adrenoceptor, L-type calcium channel, and ATP-sensitive potassium channel. The anti-hypertensive database is shown in Figure S4. Normalized fit value was the index to evaluate the overlap degree between oligopeptides and pharmacophore. As a general cutoff value in biological activity prediction, value of 0.7 was regarded as the threshold in screening process [44,45]. Oligopeptides with fit value more than 0.7 were considered as the reverse screening results.

### 3.3. Molecular Docking of ACEIs

Three crystal structures of 1O86, 4CA5 and 4BZR were utilized for the study of molecular docking by CDOCKER algorithms in DS. The common problems were automatically solved, including cleaning crystallographic waters, adding hydrogen atoms and building missing loops. The protein binding sites were determined by the initial ligands respectively. As ACE is the zinc dependent protein, Zn^2+^ should remain in the active site of ACE. Then, the initial ligands were extracted from the active site, and re-docked into the site for calculating the RMSD. RMSD less than 2.00 Å indicated docking algorithm is a reliable computational procedure and could reappear the binding of enzyme and ligands. Finally, the crystal structures with the reasonable RMSD were used for further docking with the positive ACEIs and oligopeptides from adlay. The docking results of three crystal structures were intersected. The docking score, key residues and binding poses were analyzed for selecting the potential oligopeptides [46,47]. The obtained oligopeptides were further evaluated by biochemistry assay and MD.

### 3.4. ACE Inhibitory Activity Assay

#### 3.4.1. Materials and Reagents

The potential oligopeptides obtained by docking were chemically synthesized by the Beijing Scilight Biotechnology Ltd., Co. (Beijing, China). The purity levels of the synthesized oligopeptides were all greater than 98% by HPLC analysis. ACE from rabbit lungs and HHL was supplied by Sigma-Aldrich (St. Louis, MO, USA). Acetonitrile of HPLC grade was purchased from Fisher Scientific (Pittsburgh, PA, USA). Lisinopril was supplied by Beijing HWRK Chem Co., Ltd. (Beijing, China) with more than 98% purity. All other used chemicals were of analytical grade.

#### 3.4.2. Biochemistry Assay

The assay of in vitro ACE inhibitory activity was performed by RP-HPLC method by Yuan [9]. Initially, the mixture was incubated at 37 °C for 10 min, including 10 μL of samples and 20 μL ACE (2 mU). After adding 20 μL of 2 mM HHL, the mixture continued to be incubated for 60 min until acetonitrile was added to terminate reaction. HA from the hydrolysis of the substrate HHL was detected by RP-HPLC on a C18 column (250 × 4.6 mm, 5 μm, Tianhe) at 228 nm. The column was eluted at a flow rate of 1 mL/min using 25% (*v*/*v*) acetonitrile in water with 0.05% trifluoroacetic acid. Lisinopril and borate buffer solution were used as positive and negative control, respectively. The formula (1) for computing ACE inhibitory rate is shown as follows. Wherein, *A*_control_ is the peak area of HA by negative control and *A*_sample_ is the peak area of HA by the sample. The IC_50_ was calculated by the dosage and efficiency curve based on the 50% inhibition of ACE.

(1) $$\text{Inhibitory activity}\;\left(\%\right)=\frac{A_{\text{control}}-A_{\text{sample}}}{A_{\text{control}}}\times 100\%$$

### 3.5. Molecular Dynamics Simulation

Three potential oligopeptides and positive ACEI of lisinopril were further implemented to molecular dynamics simulation. The best binding modes of 1O86–lisinopril, 1O86–NPATY, 1O86–NCHEF and 1O86–VSAIGF were selected as the start pose of MD with water environment. The MD simulations were run with GROMACS 5.0.2 using the GROMOS96 43a1 force field [48]. The topology files of ligands were generated by the GlycoBioChem PRODRG2 Server [49]. The complex systems were solvated by explicit SPC water model in cubic boxes maintaining a minimum 12 Å distance from cube edge. Eleven sodium ions were added by randomly replacing water molecules to achieve a neutral simulation cell. Each complex system was then minimized using a steepest descent integrator with 5000 steps.

A 500 ps NVT equilibration was performed at temperature of 300 K with position restraints applied to protein and ligand in order to relieve any bad contacts at the residues solvent interface [50]. Then a 1000 ps NPT simulation was conducted, and pressure was coupled to 1.0 atm using the Parrinello−Rahman method. Upon completion of the two equilibration phases, the position restraints were released and the MD was performed for 15 ns with the v-rescale method and Parrinello–Rahman method [51,52]. The snapshot structures and energies were saved for every 10 ps. The MD trajectory was determined for RMSD of 1O86 protein backbone and total energy of 1O86–ligands [53]. At the same time, RMSD of ligands and active fragments in C-terminal of oligopeptides were also analyzed for the conformational changes. Time dependent atom distances for ligands in the ACE binding site were also analyzed respectively.

## 4. Conclusions

To explore the anti-hypertensive bioactive components of adlay, in silico proteolysis and virtual screening were implemented to discover the ACE inhibitory oligopeptides. In this paper, 10 sequences of adlay prolamin were in silico hydrolyzed, and 134 oligopeptides were obtained to construct the peptide library. The reverse target identification of this peptide library was implemented based on pharmacophore, and ACE was identified as the main potential target for adlay bioactive oligopeptides. Three crystal structures of ACE were used to refine the pharmacophore results, and three oligopeptides were selected due to the score and binding mode of docking and pharmacophore. Then, ACE inhibitory activities of three oligopeptides were assayed by RP-HPLC. NPATY (IC_50_ = 61.88 ± 2.77 μM) was verified as the definite ACE inhibitory oligopeptide from adlay. According to the pharmacophore and docking models with initial ligand and oligopeptide, hydrogen bond with ALA354, hydrophobic effect with VAL518, and electrostatic network with HIS383, HIS387, HIS513 and Zn^2+^ were regarded as the key features for the ACE inhibitory peptides. MD study of NPATY was implemented to analyze the binding stability, and key residues obtained by pharmacophore and docking were found the stable binding of ACE.

This study presented the novel research method for discovering the active oligopeptides from TCM. Compared with the methods of extraction, isolation and random bioactivity assays, the combination of in silico proteolysis, virtual screening, biochemistry assay and MD study could obtain the bioactive oligopeptides from TCM rapidly and efficiently. Actually, if the absorption and metabolism of oligopeptides could be considered more, it would be more accurate to clarify the pharmacological mechanism of TCM proteins and peptides. In this paper, the oligopeptides from TCM were again identified with better biological activity. Therefore, it is necessary for TCM primary metabolites to discover their pharmacological activity in the future research.

## Figures and Tables

**Table 1:** The known bioactive peptides by in silico proteolysis derived from adlay.

No.	Bioactivity	Sequence	Reference
1	ACE inhibition	VAY, PL, SF, IF, AR, EY, AY, AF, GF, SY	[19,20]
2	Antioxidation	AY	[21]
3	Glucose uptake stimulation	VL	[22]

**Table 2:** Reverse target identification results of adlay oligopeptides.

Target	Hits	Hits (Fit Value > 0.7)
Angiotensin-I converting enzyme (ACE)	46	41
Endothelin B receptor (ETB)	28	18
Mineralocorticoid receptor (MR)	33	5
Renin (REN)	19	4
Neutral endopeptidase (NEP)	33	3
Angiotensin II type 1 receptor (AT1)	2	2
L-type calcium channel	12	1
Endothelin A receptor (ETA)	8	0
ATP-sensitive potassium channel	37	0
β-Adrenoceptor	37	0

Note: Hits are the hit number of adlay oligopeptides, and Hits (fit value > 0.7) mean the hit number of adlay oligopeptides, of which fit values are higher than 0.7.

**Table 3:** The information of ACE crystal structures and docking analysis of initial ligands.

PDB	Ligand	Resolution (Å)	Radius (Å)	RMSD (Å)	−CDOCKER ENERGY (kcal/mol)	−CDOCKER INTERACTION ENERGY (kcal/mol)	Interaction Energy (kcal/mol)
1O86	Lisinopril	2.00	10.131	0.983	106.292	120.186	−816.083
4CA5	FI	1.85	12.481	1.180	102.324	107.505	−813.721
4BZR	K-26	1.84	8.9271	0.693	156.907	123.844	−257.571

**Table 4:** The pairwise comparison of interaction residues for three crystal structures.

Ligands	1O86 and 4BZR			1O86 and 4CA5			4CA5 and 4BZR		
	HBI ^a^	EI ^b^	HI ^c^	HBI	EI	HI	HBI	EI	HI
Initial Ligands	HIS353 HIS387 TYR523 ALA354	HIS383 HIS353 HIS387 HIS513 Zn^2+^	VAL518	HIS353 TYR520 TYR523 ALA354	Zn^2+^	VAL518	HIS353 ALA356 TYR523 ALA354	Zn^2+^	VAL518 HIS410
Positive ACEIs	HIS387 ALA354	none	VAL518	ALA354 TYR523	none	VAL380 VAL518 HIS383	ALA354	none	VAL518 HIS387
Potential Oligopeptides	HIS383 HIS387	none	VAL518	ALA354 TYR523	none	HIS383	none	none	HIS387
NPATY	TYR523 ARG522 HIS387	HIS383 HIS387 Zn^2+^	VAL518	TYR523 ALA354 GLU384	HIS383 Zn^2+^	VAL518	TYR523	HIS383 Zn^2+^	VAL518

^a^ HBI represents the residues of hydrogen bond interaction; ^b^ EI represents the residues of electrostatic interaction; ^c^ HI represents the residues of hydrophobic interaction.

**Table 5:** The scores of potential adlay oligopeptides for ACE inhibitory activity.

No.	Sequence	1O86 (kcal/mol)	4BZR (kcal/mol)	4CA5 (kcal/mol)	Fit Value
1	NPATY	128.103	114.085	94.127	0.969
2	NCHEF	116.386	103.233	101.934	0.954
3	VSAIGF	99.285	96.508	104.010	0.995

## Supplementary Materials

Supplementary materials can be found at www.mdpi.com/1422-0067/17/12/2099/s1.
