# Supplementary material for: Discovery of Anti-Hypertensive Oligopeptides from Adlay Based on In Silico Proteolysis and Virtual Screening
Source: Int J Mol Sci. 2016 Dec 14;17(12):2099. doi: 10.3390/ijms17122099 (PMC5187899; doi:10.3390/ijms17122099)
Supplement: Supplementary file 1 [file ijms-17-02099-s001.pdf]

Supplementary Materials: Discovery of Anti-Hypertensive Oligopeptides from Adlay Based on In Silico Proteolysis and Virtual Screening

Liansheng Qiao, Bin Li, Yankun Chen, Lingling Li, Xi Chen, Lingzhi Wang, Fang Lu, Ganggang Luo, Gongyu Li and Yanling Zhang

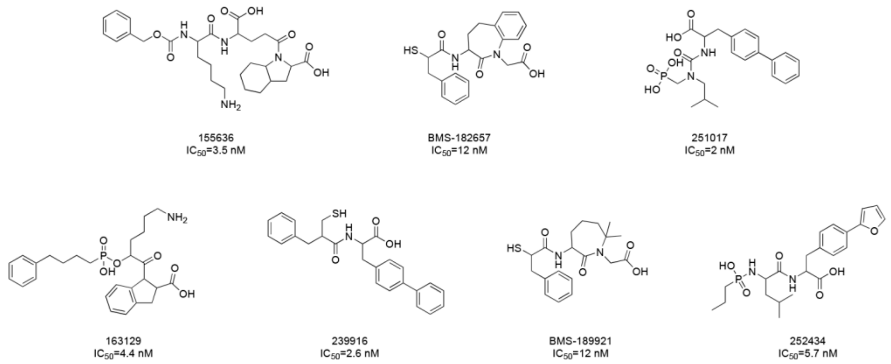

Figure S1. Training set of angiotensin-I converting enzyme (ACE) inhibitors pharmacophore. The name and IC_{50} of compounds in training set are displayed under their structure.

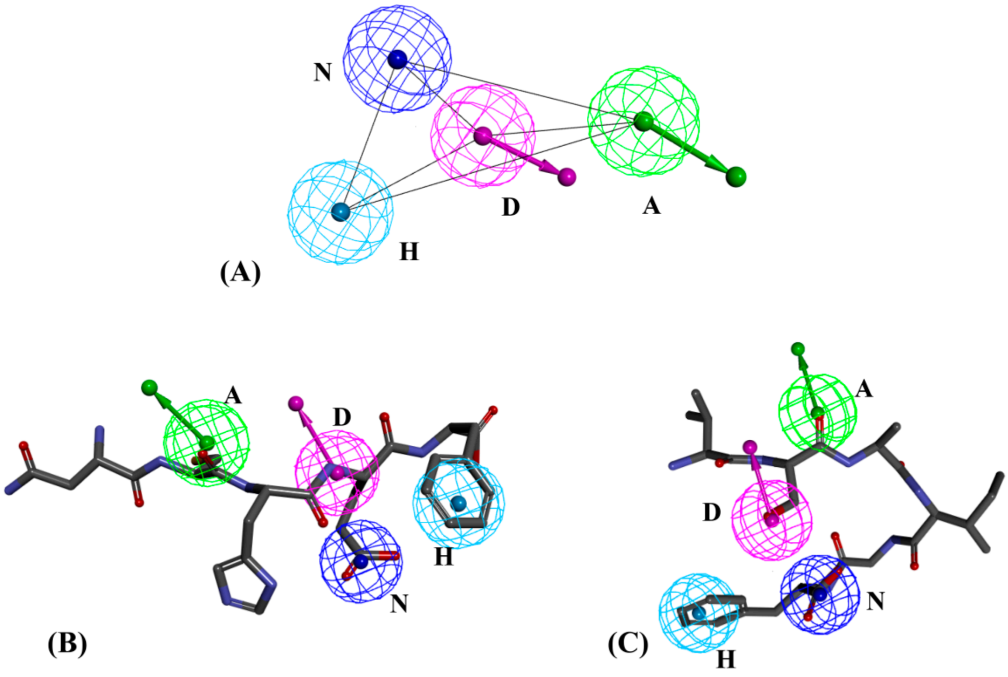

Figure S2. ACE pharmacophore model and mapping graphs: (**A**) pharmacophore model of ACE inhibitors; (**B**) pharmacophore mapping results of NCHEF; and (**C**) pharmacophore mapping results of VSAIGF. Wherein, green features represented hydrogen bond acceptor; pink features stood for hydrogen bond donor; dark blue features indicated negative ionizable group; and light blue features represented hydrophobic group.

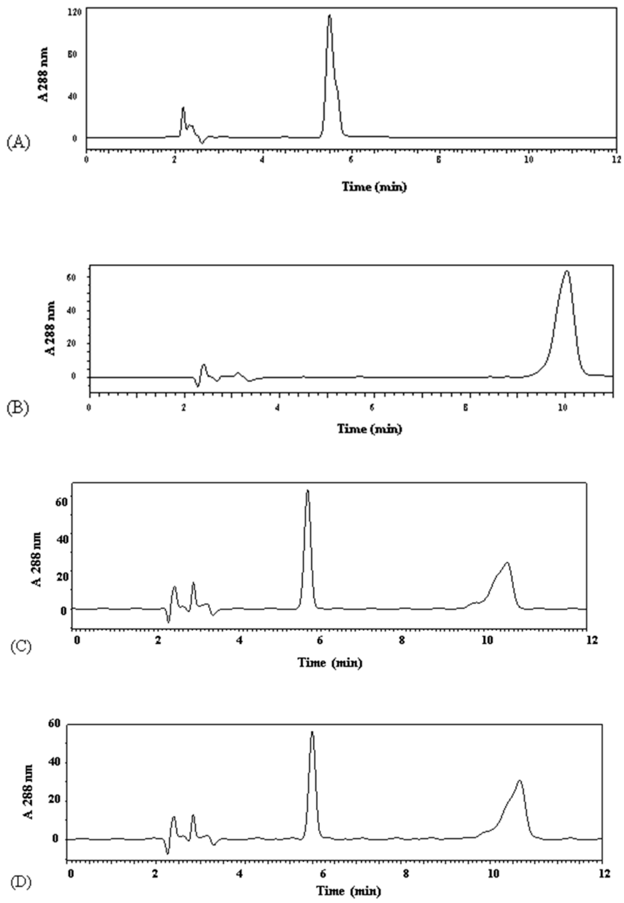

Figure S3. The reverse-phase high performance liquid chromatography (RP-HPLC) chromatograms of: (**A**) Hippuric acid (HA) standard; (**B**) hippuryl-L-histidyl-L-leucine (HHL) standard; (**C**) NCHEF; and (**D**) VSAIGF, of 0.048 mg/mL detected at 228 nm.

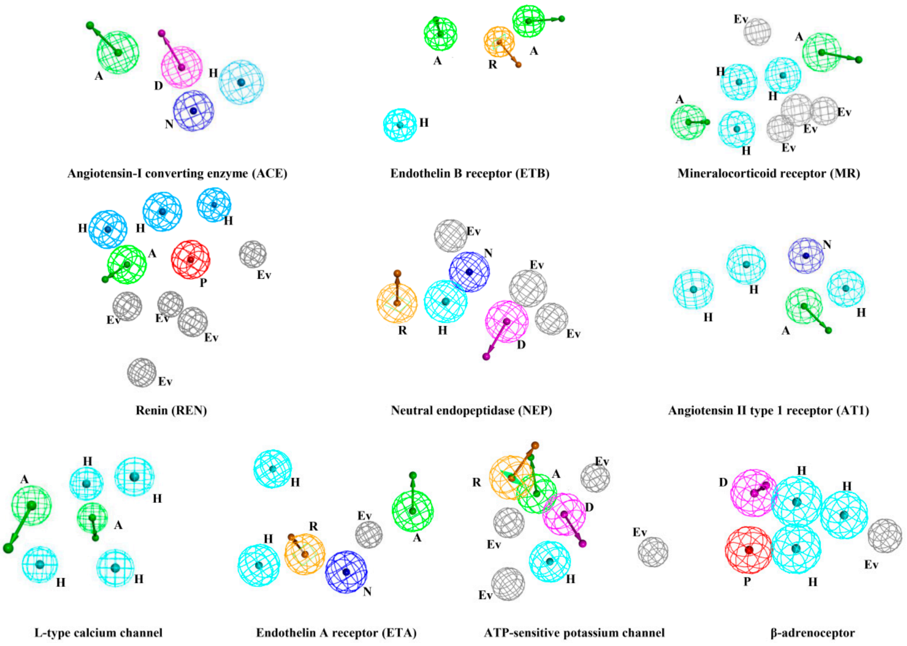

Figure S4. Anti-hypertensive pharmacophore database [1,2]. Wherein, green features represented hydrogen bond acceptor; pink features stood for hydrogen bond donor; dark blue features indicated negative ionizable group; light blue features represented hydrophobic group; orange features represented ring aromatic group; and red features stood for positive ionizable group.

Table S1. Key residues analysis of three crystal structures of ACE with diverse ligands.

Ligands	PDB	HBI	EI	HI
Initial Ligands	1O86	HIS353, HIS387, TYR520, TYR523, ALA354, GLU162, HIS513	HIS383, LYS511, HIS353, HIS387, HIS513, ZN^{2+}, TYR523	VAL518
	4BZR	HIS353, ALA356, HIS383, HIS387, GLY404, TYR523, ALA354, GLU384, SER355	ZN^{2+}, HIS383, HIS353, HIS387, HIS513, HIS410	HIS387, HIS410, TRP357, VAL518
	4CA5	GLN281, ALA356, LYS454, TYR520, TYR523, HIS353, ALA354, LYS511	ZN^{2+}, GLU376	VAL518, HIS410, HIS383, PHE527, VAL380
Positive ACEIs	1O86,	HIS383, HIS387, ALA354, TYR523	none	VAL380, VAL518, HIS383, HIS353
	4BZR	HIS387, ALA356, ALA354, SER355	none	VAL518, HIS410, TRP357, HIS387
	4CA5	ALA354, HIS353, TYR523, HIS513	none	HIS383, HIS387, VAL518, VAL380
Potential oligopeptides	1O86	HIS383, HIS387, ALA354, TYR523	none	VAL518, HIS383, ALA354,VAL380
	4BZR	HIS387, HIS383, ALA356, ARG522	none	VAL518, HIS410, TRP357, HIS387
	4CA5	ALA354, HIS353, TYR523, GLU384	none	VAL380, HIS383, HIS353, HIS387
NPATY	1O86	HIS383, GLU384, HIS387, HIS513, ARG522, TYR523, ALA354, ASP377, GLU376	GLU162, HIS383, HIS387, HIS513, ZN^{2+}	VAL518
	4BZR	ARG522, TYR523, SER516, HIS387, PRO519	HIS383, HIS387, ZN^{2+}	VAL518
	4CA5	GLN281, TYR523, ALA354, GLU384	ZN^{2+}, ASP415, HIS383	VAL518
NCHEF	1O86	GLN281, ALA356, HIS383, GLU384, HIS387, TYR523, ALA354, GLU162, GLU376, HIS353, SER355	GLU162, GLU376, ZN^{2+}	HIS383, VAL380
	4BZR	ALA356, HIS387, ARG522, ALA354, ASN70, SER355, ASP358, TRP59	GLU403, HIS387, ALA63	ZN^{2+}, HIS387
	4CA5	GLN281, LYS454, TYR520, HIS353, ALA354, GLU384, THR282	GLU411, ZN^{2+}, HIS387	VAL380
VSAIGF	1O86	HIS353, HIS383, GLU384, HIS387, LYS511, HIS513, GLU411	ZN^{2+}	ALA354, VAL380, VAL518, HIS353, TRP357, HIS383, PHE512
	4BZR	TYR360, HIS387, ARG522, SER355, ALA356, GLU411	GLU403, ZN^{2+},ARG522	TRP357, TYR360, PHE391
	4CA5	LYS511, TYR523, ALA354, HIS353, ALA356, GLU384	GLU411, ZN^{2+}, HIS410	ALA354, VAL380, HIS353, HIS383

[a] HBI represents the residues of hydrogen bond interaction; [b] EI represents the residues of electrostatic interaction; [c] HI represents the residues of hydrophobic interaction.

Table S2. The scores of screening results of 19 adlay oligopeptides.

No.	Sequence	1O86 (kcal/mol)	4BZR (kcal/mol)	4CA5 (kcal/mol)	Fit Value
1	VNPAYY	129.104	112.878	112.193	0.987
2	NPATY	128.103	114.085	94.127	0.969
3	ANPAYY	122.459	146.717	109.623	0.971
4	PCCAF	121.798	104.901	83.669	0.964
5	NCHEF	116.386	103.233	101.934	0.954
6	VMPF	114.800	94.894	71.380	0.964
7	PNNPY	112.012	103.265	64.566	0.963
8	PAAY	111.354	89.640	78.759	0.965
9	QPF	106.274	84.562	74.196	0.910
10	AQTVAF	104.258	99.967	106.895	0.937
11	TAAQY	103.702	95.323	107.942	0.948
12	HQMIY	103.514	95.549	92.492	0.937
13	VSAIGF	99.285	96.508	104.010	0.995
14	QPY	99.044	91.048	73.372	0.964
15	QQQQY	97.239	112.184	106.204	0.952
16	TATGF	96.705	96.906	101.688	0.945
17	SPF	96.625	80.108	67.720	0.971
18	SQQF	92.291	83.864	101.275	0.949
19	QQQF	89.407	97.122	100.767	0.949

Reference

1. Zhang, Y. Study on the Methodology of Chinese Medicine Virtual Screening Based on Pharmacophores. Ph.D. Thesis, Beijing University of Chinese Medicine, Beijing, China, 30 June 2006.
2. He, Y. Discovery of Potential Repositioning TCM Ingredients for Anti-Hypertension. Master's Thesis, Beijing University of Chinese Medicine, Beijing, China, 30 June 2015.
